# The Gain Modulation by N-methyl-D-aspartate in the Projection Neurons of Robust Nucleus of the Arcopallium in Adult Zebra Finches

**DOI:** 10.1155/2012/931780

**Published:** 2012-05-20

**Authors:** Su-Qun Liao, Guo-Qiang Hou, Xuan Pan, Cong-Shu Liao, Dong-Feng Li

**Affiliations:** ^1^Key Laboratory of Ecology and Environmental Science in Higher Education of Guangdong Province, School of Life Sciences, South China Normal University, Guangzhou 510631, China; ^2^School of Education, Shaoguan University, Shaoguan 512000, China

## Abstract

The song of zebra finch is stable in life after it was learned successfully. Vocal plasticity is thought to be a motor exploration that can support continuous learning and optimization of performance. The activity of RA, an important pre-motor nucleus in songbird's brain, influences the song directly. This variability in adult birdsong is associated with the activity of NMDA receptors in LMAN-RA synapses, but the detailed mechanism is unclear. The control of gain refers to modulation of a neuron's responsiveness to input and is critically important for normal sensory, cognitive, and motor functions. Here, we observed the change of gain in RA projection neurons after exogenous NMDA was applied to activate NMDA receptors using the whole-cell current clamp recording. We found that NMDA substantially increased the slope (gain) of the firing rate-current relationship in RA projection neurons. The AMPA receptor-dependent excitability played a crucial role in the modulation of gain by NMDA. These results suggested that NMDA receptors may regulate the dynamics of RA projection neurons by input-output gain.

## 1. Introduction

Song is a complex, learned motor skill that involves the precise coordination of vocal and respiratory musculature in order to produce highly stereotyped renditions of a memorized song model. Two pathways contributed to this behavior are identified in the songbird forebrain: the motor pathway that is required throughout life for normal song production, and the anterior forebrain pathway (AFP) which is necessary for song learning and plasticity [1–3]. HVC (used as the proper name) is the beginning nucleus of both pathways and projects to the robust nucleus of the arcopallium (RA) and AFP. The motor pathway is from HVC to RA, and then RA projects to brainstem nuclei controlling the vocal and respiratory muscles [4]. During song, RA neurons in adult birds generate a highly stereotyped sequence of bursts [5, 6] and also receive input from lateral magnocellular nucleus of the anterior nidopallium (LMAN), the output nucleus of AFP. Notably, recordings from LMAN neurons projecting to RA revealed highly variable spiking activity across song renditions, suggesting that LMAN may act as a source of variability. Blockade of synaptic inputs from LMAN to RA with TTX reduced song variability [7]. These results indicate that synaptic activity in LMAN-RA synaptic transmission is crucial to song plasticity [8, 9], but the detailed mechanism is unclear.

It has been observed that RA received excitatory glutamatergic inputs from HVC and LMAN. The former is mediated by a mixture of N-methyl-D-aspartate (NMDA) and *α*-amino-3-hydroxy-5-methyl-4-isoxazolepropionic-acid- (AMPA-) type receptors [4, 10, 11], and the latter is mediated almost exclusively by NMDA-type receptors [11]. LMAN-RA synaptic transmission is necessary for the variability of song in adult zebra finch [8], suggesting that the activity of NMDA receptor on the LMAN-RA synapses might induce the activity pattern change of RA neurons and change the bursts that control the vocal and respiratory muscles [4].

The control of gain, which refers to modulation of a neuron's responsiveness to input, is critically important for normal sensory, cognitive, and motor functions [12–15]. Gain control is achieved through a divisive process and is observed as a change in the slope of the input-output relationship. Such gain changes are frequently observed in the responses of cortical neurons and are thought to play an important role in neural computations [14]. Modulations of gain have been observed in the enhancement of neural responses by attention [12, 16] and can also be induced pharmacologically [17]. In a neuron model, NMDA receptors caused such membrane potential fluctuations between a hyperpolarized down-state and a depolarized up-state, which may gate the information and gain modulation [18]. Here, we test the gain change of RA projection neurons after exogenous NMDA was applied to selectively activate NMDA receptors, using whole-cell current clamp recording in adult male zebra finch slices.

## 2. Material and Methods

### 2.1. Slice Preparation

Brain slices were prepared from adult male zebra finches (*Taeniopygia guttata*) (>90 days old) obtained from commercial suppliers as previously described [19, 20]. All experiments were carried out in accordance with the university and national animal guidelines. Briefly, birds were anesthetized with 10% chloral hydrate and were then rapidly decapitated. Brains were dissected into ice-cold, oxygenated (95% O_2_ and 5% CO_2_) slice solution. Slice solution consists of (in mM) sucrose 248, KCl 5, NaHCO_3 _ 28, glucose 10, MgSO_4_·7H_2_O 1.3, and NaH_2_PO_4_·H_2_O 1.26 [21, 22]. Coronal brain slices (250 *μ*m thick) containing RA were cut with a vibrating microtome (MA752, USA) and collected in artificial cerebrospinal fluid (ACSF) that had been warmed to 37°C and subsequently allowed to cool to room temperature. Slices were allowed to recover in the holding chamber for at least 1.5 h and were equilibrated to room temperature before recordings were made. Standard ACSF consists of (in mM) NaCl 125, NaHCO_3_ 25, NaH_2_PO_4_·H_2_O 1.27, KCl 2.5, MgSO_4_·7H_2_O 1.2, CaCl_2 _ 2.0, and glucose 25 and was adjusted with sucrose to a final osmolarity of 350 mOs [21].

### 2.2. Patch-Clamp Recording

For recording, a slice was transferred to a submerge-type chamber where it was continuously exposed to ACSF, saturated with 95% O_2_ and 5% CO_2_ at room temperature (22–26°C) at the rate of 2.0 mL/min. RA and the surrounding tissues were distinguishable under BX51WI microscope connected with a DIC-IR video camera (Olympus, Japan). At high magnification (40x), RA neurons were visualized and the recordings were made from healthy cells. Recording pipettes were fabricated from borosilicate glass (Sutter Instruments, USA) using a Flaming-Brown puller (model P-97, Sutter Instruments, USA) and were filled with the pipette solution containing (in mM): K MeSO_4 _ 120, NaCl 5, HEPES 10, EGTA 2, Mg-ATP 2, and Na_3_-GTP 0.3 (pH 7.3-7.4, and 340 mOs). The recording pipettes, which had resistances ranging from 3 to 5 MΩ, were positioned using an integrated motorized control system (Sutter Instruments, USA). Tight-seal whole-cell recordings were obtained using standard techniques. The potential changes after application of NMDA were corrected during the input-output stimulation protocols. The baseline membrane potential was set at −60 mV during those stimulation protocols. The series resistance (*R*_*s*_), between 15 MΩ and 30 MΩ, was compensated using the bridge balance.

### 2.3. Drugs and Drug Delivery

All agents were applied by changing the bath perfusate from standard ACSF to modified ACSF in which various drugs were simply added. Unless indicated otherwise, all solutions were continuously bubbled with 95% O_2_ and 5% CO_2_. All chemicals were purchased from Sigma.

### 2.4. Data Analysis

The Clampfit 9.2 (Axon Instruments, USA), Mini 6.0 (Synaptosoft Inc., Fort Lee, NJ, USA), and OriginPro 8.0 (OriginLab, Nothampton, MA, USA) were used for analysis. Action potentials were detected using the event detection package of the Clampfit 9.2. Events with peak amplitude of 50 mV or higher and a rise time of about 0.5 ms were detected automatically, and the results were analyzed with Origin Pro 8.0. The steady-state spike rate was estimated by counting the number of spikes in 1 second, and the result was plotted versus the intensity of the injected current (f-I relationship). There were no differences in threshold current values between different groups of neurons. The slope of the f-I relationship (referred to as gain) was estimated by linear fitting. Slope parameters were estimated separately for individual neurons and that Figures 4 and 5 contain mean slope values averaged for the whole groups of neurons. Input resistance was estimated by applying small hyperpolarizing current pulses. The amplitude of AHP was quantified as the difference between spike threshold and the lowest point of AHP. Throughout, means ± SD are given. Means were compared using paired two-tailed Student's *t*-test or one-way ANOVA followed by Scheffe's multiple comparison.

## 3. Results

RA neurons were observed under DIC-IR optics. Stable whole-cell recordings were obtained in 160 RA projection neurons from 51 adult male zebra finches. A neuron with the resting membrane potential <−50 mV and an overshooting action potential was considered viable. Neurons that did not meet these criteria were discarded. RA projection neuron was identified by a larger soma, time-dependent inward rectifier induced by hyperpolarizing current and spontaneous regular spike firing or by depolarizing current pulses [23].

### 3.1. Gain Modulation by NMDA in RA Projection Neurons

**Figure 1:**
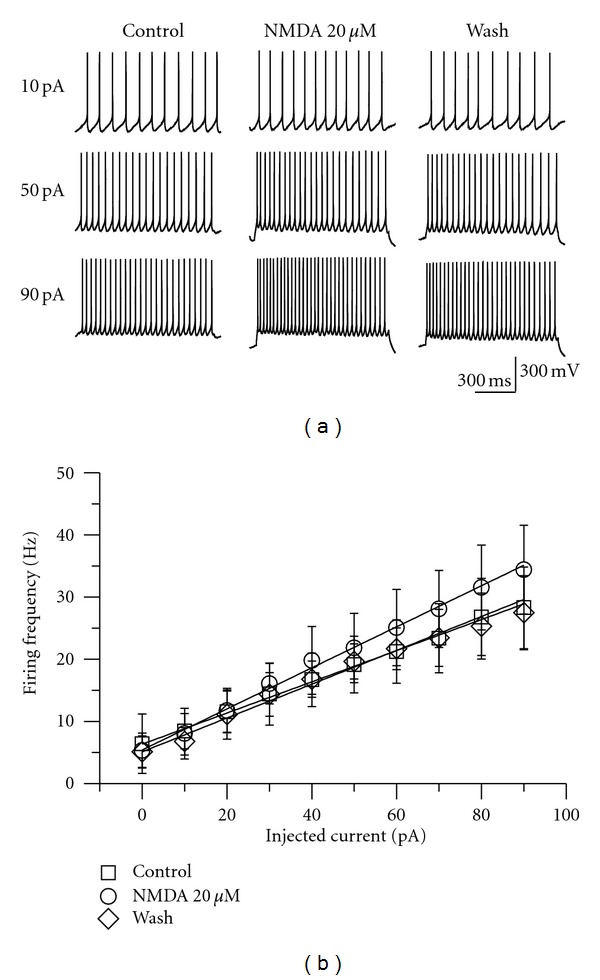
(a) Traces of action potentials in response to 1 s current steps of 10, 50, and 90 pA (top to bottom). The average frequencies were 8.98, 18.54, and 27.05 Hz, respectively, before NMDA application (left column); NMDA increased the frequency and the average frequencies were 8.72, 23.62, and 33.34 Hz after application of NMDA (20 *μ*M) for 8 min (middle column); the effects were reversed after wash for 15 min (right column). (b) The slope of the f-I relationships was changed after 20 *μ*M NMDA application when depolarizing current steps from 0 to 90 pA. The slopes were 246.92 ± 28.00, 325.49 ± 73.34, and 253.03 ± 48.54 Hz/nA for the control, NMDA treatment, and wash, respectively (*P* < 0.05, *n* = 11). 20 *μ*M NMDA increased the gain of RA projection neurons. The average in control is shown as open squares, group in NMDA is shown as open circles, and the group after wash is shown as open diamonds.

**Figure 2:**
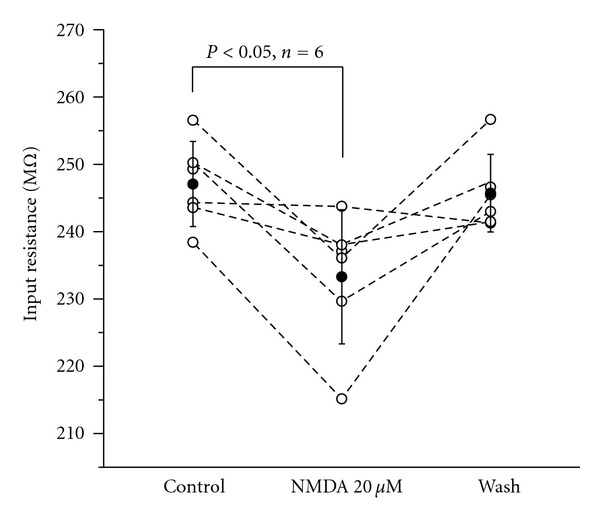
A decrease of input resistance induced by NMDA in RA projection neurons. The input resistance was decreased to 233.29 ± 9.97 MΩ after application of 20 *μ*M NMDA from 247.07 ± 6.31 MΩ and was recovered to 245.72 ± 5.75 MΩ after wash for 10 min. Individual cells are shown as open circles, and the average of each group is shown as an closed circle. *P* < 0.05 (*n* = 6, paired *t*-test).

The input-output relationship was examined by injecting current pulses from 0 to 90 pA with an increment of 10 pA and an interval of 15 s, and they were lasted for 1 s. Traces of action potentials in response to 1 s current steps of 10, 50, and 90 pA were shown in Figure 1(a). The slopes of the f-I relationships were 246.92 ± 28.00 Hz/nA before NMDA application and increased to 325.49 ± 73.34 Hz/nA after NMDA application—an increase of 131.99% of the control. The slope returned to 253.03 ± 48.54 Hz/nA after 15 min of washing (Figure 1(b)). Furthermore, the input resistances were decreased by 20 *μ*M NMDA in RA projection neurons (*P* < 0.05, *n* = 6) (Figure 2). The activation of NMDA receptors may lead other ion channels open. Thus, these results showed that NMDA had an effect on the slope.

**Figure 3:**
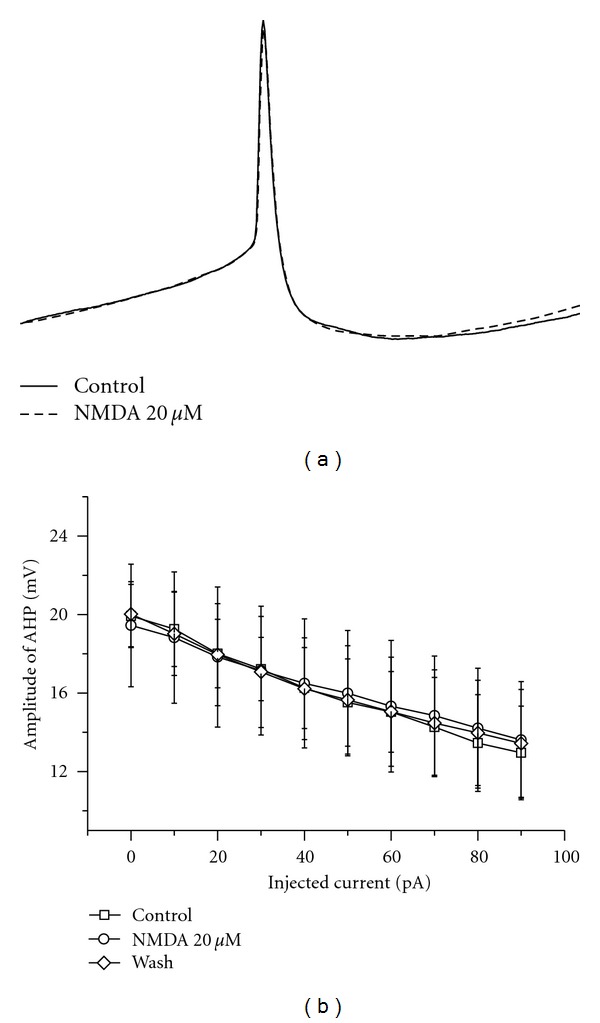
The effect of NMDA on the spike AHP in RA projection neurons. (a) Action potentials evoked by 90 pA current injection are overlaid to compare the change of AHP by NMDA application. There is no obvious change in spike shape. The spike in control is shown as a solid line, and that in NMDA is shown as a dash line. (b) Plot of amplitude of AHPs versus injected current. NMDA did not cause a significant change in average amplitude of AHPs (*n* = 5). Paired *t*-test was used to evaluate the differences between the groups.

The afterhyperpolarization (AHP) of action potential underlies the modulation of firing frequency. To test the effect of NMDA on spike AHP, we analyzed the change of spike AHP before and after NMDA application. NMDA did not lead to an obvious effect on spike AHP. Comparison of spike shape is shown in Figure 3(a) with 90 pA current injection before and after NMDA application. In average, there was no significant change in the amplitude of AHP after NMDA application (*P* > 0.05, *n* = 5) (Figure 3(b)).

**Figure 4:**
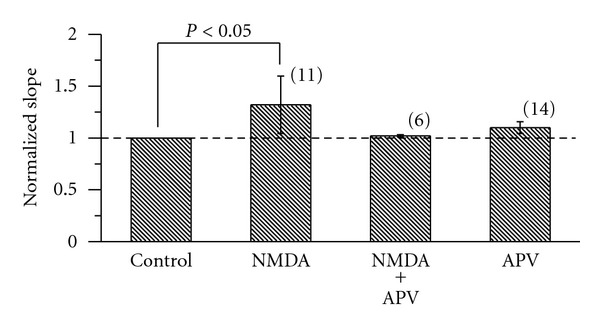
The effect of DL-APV on the gain induced by NMDA in RA projection neurons. The f-I slopes of NMDA (20 *μ*M) alone, NMDA (20 *μ*M) +DL-APV (50 *μ*M), and DL-APV (50 *μ*M) alone were normalized to control, respectively. The broken line indicates the level of control (100%). The number of cells in each group is given in parentheses. *P* < 0.05 (One-way ANOVA followed by Scheffe's multiple comparison).

Then we used 50 *μ*M DL-APV, a NMDA receptor antagonist, to check the selectivity of NMDA receptor,and we found that the slope of f-I was not changed by DL-APV alone (*P* > 0.05). The slope was 255.78 ± 66.69 Hz/nA and 269.34 ± 56.96 Hz/nA (*P* > 0.05) before and after application of 50 *μ*M DL-APV (Figure 4), respectively. NMDA increased the f-I slope 1.32 times (*P* < 0.05); however, the gain modulation induced by NMDA was completely abolished by DL-APV (Figure 4).

### 3.2. The Role of Other Neurotransmitters on f-I Slope

**Figure 5:**
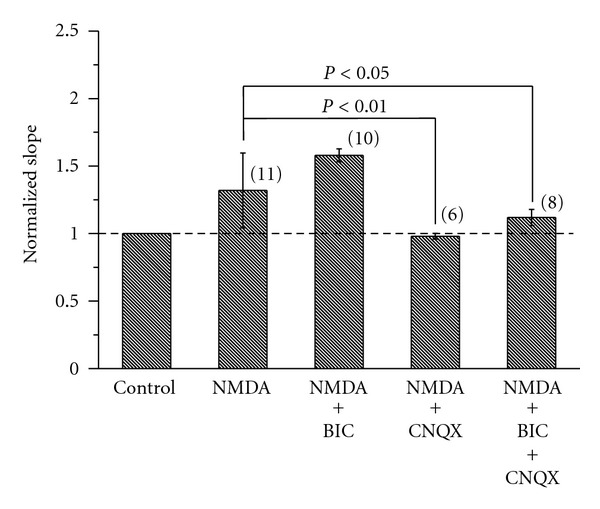
The effect of CNQX on the gain induced by NMDA in RA projection neurons. The results were obtained with NMDA (20 *μ*M), NMDA (20 *μ*M) in the presence of bicuculline methiodide (BIC, 10 *μ*M), NMDA (20 *μ*M) in the presence of CNQX (10 *μ*M), NMDA (20 *μ*M) in the presence of BIC (10 *μ*M), and CNQX (10 *μ*M). The slopes were normalized to those of controls. The broken line indicates the level of control (100%). The number of cells in each group is given in parentheses. *P* < 0.05 or *P* < 0.01 (one-way ANOVA followed by Scheffe's multiple comparison).

Previous studies reported that RA projection neurons received glutamatergic and GABAergic inputs [23, 24], which might increase background synaptic activities and modulate the gain of neurons [25, 26]. To determine the effect of AMPA receptor and GABA receptor on gain modulation by NMDA, we examined the effects of NMDA in the presence of CNQX, an antagonist for ionotropic glutamate AMPA receptors, or/and bicuculline methiodide (BIC), a competitive antagonist of GABA_A_ receptors, respectively. BIC (10 *μ*M) accelerated the depolarization by NMDA application, but BIC alone had little effect on the f-I slope. In the presence of BIC, the mean initial slopes were 276.46 ± 59.4 and 332.6 ± 40.02 Hz/nA before and during application of NMDA. The f-I slope was increased from 1.32 to 1.39 in the presence of NMDA and BIC (*P* > 0.05); that is, there is an increase of 105.30% over the NMDA treatment (Figure 5; *P* > 0.05, unpaired *t-*test). CNQX (10 *μ*M) decreased the membrane potential and abolished the spontaneous spikes of RA projection neurons sometimes but did not affect the slope of f-I relationship. In the presence of CNQX, NMDA application hardly changed the f-I slope of RA projection neurons no matter whether BIC was present or not (Figure 5; *P* > 0.05). These results indicated that the AMPA receptor-dependent excitability played a crucial role in the gain modulation by NMDA. The slope was 0.97 or 1.11 in the presence of CNQX or CNQX and BIC, respectively. The gain induced by NMDA was significantly decreased by CNQX (*P* < 0.05).

## 4. Discussion

In this study, we showed that NMDA increased the gain of RA projection neurons in adult zebra finch. NMDA-induced gain increase was limited to firing frequencies less than 40 Hz. The effect of NMDA on gain was mediated through NMDA receptors and might involve synaptic activities.

### 4.1. Effects of NMDA on the Gain of Neurons

In songbirds, we found that NMDA increased the slope of f-I relationship in RA projection neurons, which was completely blocked by DL-APV, a NMDA receptor selective antagonist, suggesting that NMDA increased the sensitivity of RA projection neurons. The pharmacology of NMDA receptors involved in gain modulation has been examined in mammals. In cat visual cortex, NMDA increased the gain of the contrast-response (C-R) curve of the neuron in layers II/III and V/VI [17]. In mouse, the thalamocortical neurons in the dorsal lateral geniculate nucleus play a key role in the generation of firing patterns through NMDA receptors [27].

The neural activity in the AFP of songbirds can direct moment-by-moment changes in the primary motor areas responsible for generating song. LMAN is the last output nucleus of AFP. Singing-related activity in LMAN is more variable in pattern across repeated renditions during undirected song than directed song [28, 29]. Kao et al. reported that the fundamental frequency of the syllable was shifted by stimulating the LMAN-RA pathway, which is mediated by NMDA receptors [29]. Our results indicated that the activity of NMDA receptor changed the sensitivity of RA projection neuron. RA, as a premotor nucleus, occupies an important position in the song system, integrating information from both pathways. RA projection neurons are responsible for transmitting song information to midbrain and brainstem vocal and respiratory structures [30]. Temporal pattern of spike bursts in RA projection neurons is associated with the timing of acoustic features of birdsong. Precise timing of individual spikes has a close relationship with stereotypic behavior, which suggests that the song is represented in RA by a temporal code [5, 31]. Thus, the modulation of NMDA receptors in the activities of RA projection neurons could be very important for the plasticity of birdsong.

### 4.2. The Possible Mechanism of the Gain Modulation by NMDA

Previous studies in slices demonstrated that NMDA receptor achieves the nonlinear, multiplicative behavior seen in gain modulation by several mechanisms. Mel presented results from compartmental modeling studies showing that dendrites containing NMDA-type synaptic conductances and voltage-dependent Na^+^, K^+^, and Ca^2+^ channels can impose nonlinear interactions between nearby synapses that give rise to approximate multiplications [32, 33]. In thalamocortical cell of the rat and cat dLGN, NMDA receptors contribute to the burst firing generated by the low-threshold Ca^2+^ potential and depend on the membrane potential range over which this type of firing occurs [34]. In substantia nigra dopamine neurons, NMDA receptor-mediated synaptic conductance generates transient high-frequency activity by rapidly but transiently overwhelming the conductance's underlying action potential AHP and/or engaging postsynaptic voltage-dependent ion channels in a manner that overcomes the limiting effects of AHP [35]. But our results showed that there was no significant change in spike AHP after NMDA application. So the gain induced by NMDA in RA projection neurons may not be mainly modulated by the change of spike shape, but by synaptic activities.

The change in membrane potential has been shown to play an important role in the firing pattern regulation by NMDA receptor [27], supporting the idea that depolarization acutely alters firing frequency. It has been shown that simple excitability or inhibition alone can induce a multiplicative gain change in the cortical neurons. In this model, one set of synaptic inputs provided purely excitatory drive to a target neuron, and another set of combined excitatory/inhibitory inputs (or balanced inputs) provided the modulation. In this setup, changing the firing rates of the balanced inputs produced an approximately multiplicative gain modulation of the response to the purely excitatory drive [14]. Here, blockade of the excitatory or inhibitory transmission alone had no effect on the f-I slope of RA projection neuron, implying that simple excitability or inhibition alone cannot induce a multiplicative gain change. However, our results did not exclude a role of spontaneous synaptic activity on gain modulation *in vitro*, which, due to the loss of synapses in slice, was not sufficient to produce a significant change in gain. 

AMPA receptors are required for the gain modulation in our study, because blocking excitatory transmission mediated by AMPA receptors almost abolished the effect of NMDA on gain modulation. Two reasons might be involved: one is the two states of NMDA-base mechanism due to the special anatomical structure of RA. RA projection neurons receive inputs from two areas, HVC and LMAN. Both are excitatory glutamatergic but have distinct postsynaptic properties. HVC-RA is largely mediated by AMPA receptor, whereas LMAN-RA is almost completely mediated by NMDA receptor [10]. AMPA/NMDA receptor composition of different neuronal pathways is the foundation of gating and gain modulation. NMDA receptor-rich pathway can gate AMPA-rich input and increase the gain of a neuron responding to the input of AMPA-rich pathway [18]. Thus, AMPA receptors are required for the gain modulation by NMDA receptors. The second is related to the intrinsic properties of NMDA receptors, which are uniquely voltage dependent. Except binding with neurotransmitter, the activation of NMDA receptors also needs depolarization of postsynaptic membrane potential. Blockade of main excitatory inputs mediated by AMPA receptor with CNQX hyperpolarized the membrane potential, which caused NMDA receptors not activated. Therefore, the AMPA receptor-mediated excitatory transmission is essential for the gain modulation induced by NMDA.

### 4.3. Functional Implications of NMDA-Induced Gain Modulation

It is well known that the NMDA receptor plays a critical role in many physiological and pathological processes, such as learning, memory, neurodegeneration, epilepsy, and ischemia. The NMDA receptor-mediated synaptic transmission satisfies the associative property of Hebbian learning and in fact plays a critical role in its cellular model, long-term synaptic plasticity. Nevertheless, pharmacological agents which block NMDA receptors impair a variety of brain processes, suggesting that NMDA receptor transmission also plays an important role beyond long-term memory and participates in shaping the dynamic activity of neural networks [36]. In song system, the expression pattern of NMDA receptor is related to the development of song learning [21, 37–40]. Recent results show that the NMDA receptor on the LMAN-RA synapses generates variability across different song renditions, thereby facilitating reinforced learning of songs. How NMDA accomplishes this role is not clear. Our results showed that NMDA application increased the gain of RA projection neurons, in which AMPA receptor was necessary. The AMPA receptor-mediated excitability is derived only from HVC [10, 11]. Therefore, NMDA receptor in LMAN-RA synapse might regulate HVC-RA synaptic transmission, which implicated that NMDA receptor is more effective to modulate the syllable online. These findings suggest a mechanism by which NMDA receptor can selectively modulate behaviorally relevant excitatory inputs.
